# Effects of Functional Training on Body Composition in Adults with Overweight or Obesity: A Systematic Review and Meta-Analysis of Randomized Clinical Trials

**DOI:** 10.3390/jfmk11030275

**Published:** 2026-07-17

**Authors:** Ana Carolina Arruda Meira Brito, Alan Bruno Silva Vasconcelos, Laíza Ellen Santana Santos, Paulo Ricardo Martins-Filho, Marzo Edir Da Silva-Grigoletto

**Affiliations:** 1 Department of Medicine, Federal University of Campina Grande, Campina Grande 58429-900, PB, Brazil; 2 Department of Health Education, Federal University of Sergipe, Lagarto 49400-000, SE, Brazil; absvasconcelos@gmail.com; 3 Postgraduate Program in Health Sciences, Federal University of Sergipe, Aracaju 49107-230, SE, Brazil; laizaaellen@gmail.com (L.E.S.S.); dasilvame@gmail.com (M.E.D.S.-G.); 4 Investigative Pathology Laboratory, Federal University of Sergipe, Lagarto 49400-000, SE, Brazil; prmartinsfh@gmail.com; 5 Department of Physical Education, Federal University of Sergipe, São Cristóvão 49107-230, SE, Brazil

**Keywords:** exercise, body composition, health

## Abstract

**Background:** Obesity is a chronic condition that compromises health and quality of life, with physical exercise representing a relevant therapeutic strategy in its management. Functional training (FT) has been adopted as a modality that integrates neuromuscular and cardiorespiratory stimuli, promoting both physical and psychosocial benefits. However, gaps remain in the literature regarding the effects of FT on body composition in individuals with excess weight. In this context, a systematic review was conducted to analyze the current state of scientific knowledge on this approach. **Methods:** Randomized controlled clinical trials investigating FT were selected, including interventions classified as high-intensity functional training (HIIT-F), multicomponent programs, and CrossFit-like interventions when they fulfilled the predefined FT eligibility criteria. Participants were required to be between 18 and 65 years of age, physically independent, and in generally good health. The systematic search was conducted in the PubMed/MEDLINE, Embase, Web of Science, Cochrane Library, PEDro, and LILACS databases. The risk of bias (RoB 2.0) and the certainty of the evidence (GRADE) were assessed. **Results:** A total of 2,972 records were identified. Seven studies met the eligibility criteria, comprising 296 participants. Two randomized controlled trials (61 participants) were eligible for meta-analysis. The pooled analysis demonstrated a significant reduction in body fat percentage (%BF) in favor of FT/HIIT-F compared with non-exercising controls (mean difference = −4.44 percentage points; 95% confidence interval −7.08 to −1.80; *p* = 0.001). Risk of bias assessment showed moderate methodological limitations across studies. None were classified as low risk. Three had high risk due to missing outcome data and deviations from intended interventions, whereas four showed some concerns related to allocation concealment and analysis reporting. Bias in outcome measurement was consistently low. **Conclusions:** Current evidence suggests that FT/HIIT-F may be associated with reductions in %BF among adults with overweight or obesity. However, these findings should be interpreted with caution because the certainty of the evidence was rated as very low according to the GRADE approach, the quantitative synthesis included only two studies, and important methodological limitations were identified. As the available data are predominantly derived from female participants, the applicability of these findings to men remains uncertain.

## 1. Introduction

Obesity is a chronic and progressive condition, widely recognized as a global health epidemic, and is characterized by excessive accumulation of body fat [1,2]. This accumulation significantly increases the risk of chronic non-communicable diseases, such as type 2 diabetes, hypertension, and cardiovascular diseases, while negatively impacting quality of life and longevity [1,2].

Effective obesity treatment requires a multidisciplinary approach focused on sustainable lifestyle changes, including dietary re-education, regular physical activity, and psychological support. Although exercise alone may be less effective for initial weight loss than energy restriction, it plays a crucial role in maintaining long-term weight loss and improving overall metabolic health [3,4]. Additionally, physical exercise helps preserve lean mass (LM) during weight loss by mitigating the catabolic effects of restrictive diets, while simultaneously enhancing physical function and resting energy expenditure. Accordingly, physical activity constitutes an essential component of any comprehensive therapeutic strategy for managing obesity [5].

Several forms of physical exercise have been recommended for obesity management, including aerobic training (AT), resistance training (RT), combined training (CT), and high-intensity interval training (HIIT), each with distinct physiological benefits for improving body composition and supporting weight regulation [6]. In this context, FT has emerged as a promising and integrative approach to health promotion and physical conditioning. FT combines neuromuscular and cardiorespiratory stimuli with proprioceptive, agility, and coordination exercises, offering a multifaceted strategy to improve executive function performance and muscular endurance [7,8].

Although FT is often presented as a distinct exercise modality, its conceptual boundaries overlap with multicomponent training, CrossFit-like programs, and HIIT-F. According to a recent international consensus, FT can be defined as a physical interventional approach aimed at enhancing human performance according to individual goals, while considering task specificity and individual responsiveness [9]. From this perspective, training programs may be better understood along a continuum of functionality rather than as rigidly separated categories. Thus, in the present review, multicomponent programs, CrossFit-like interventions, and functional forms of HIIT were considered within the scope of FT when they incorporated multi-joint movement patterns, integrated multiple physical capacities, and aimed to produce transferable adaptations relevant to activities of daily living [9].

By integrating these elements, FT promotes improvements in motor mobility, functional capacity, and overall physical fitness. These characteristics reinforce its potential as a relevant exercise modality for individuals with overweight or obesity, particularly due to its ability to simultaneously stimulate multiple physical capacities that may influence body composition outcomes [10].

Despite the growing interest in FT as a strategy for obesity management, important knowledge gaps remain concerning its therapeutic efficacy, especially in relation to changes in body composition. Therefore, this systematic review aims to synthesize and critically evaluate the current evidence on the effects of FT on body composition parameters in adults with overweight or obesity.

## 2. Materials and Methods

### 2.1. Protocol and Registration

The protocol of this systematic review was previously registered in the International Prospective Register of Systematic Reviews (PROSPERO) (protocol number CRD42024564492). The review was conducted in accordance with the guidelines of the Cochrane Collaboration for systematic reviews and meta-analyses [11] and reported following the Preferred Reporting Items for Systematic Reviews and Meta-Analyses (PRISMA) statement [12].

### 2.2. Research Question

This systematic review aimed to address the following research question: “Does functional training promote changes in body composition in adults with excess weight?”

### 2.3. Eligibility Criteria

Inclusion and exclusion criteria were defined based on the PICOS framework (Population, Intervention, Comparison, Outcomes, and Study Design). Studies were included if they met the following criteria:

• Population (P): adults (aged 19–64 years), of any sex or ethnicity, classified as overweight or obese according to the World Health Organization (WHO) criteria (Body Mass Index—BMI ≥ 25 kg/m^2^) [13]. Studies including individuals with comorbidities commonly associated with excess weight (e.g., hypertension, type 2 diabetes) were included if the primary condition did not directly impair motor function.
• Intervention (I): FT protocols consisting multi-joint, integrated exercises that mimic daily movements and incorporate strength, power, balance, agility, or coordination. Studies were excluded if the intervention was inadequately described or could not be clearly identified as FT.
• Comparator (C): control group (no exercise) or another structured exercise modality (e.g., aerobic training, resistance training).
• Outcome (O): body composition outcomes assessed using validated methods, such as dual-energy X-ray absorptiometry (DXA), bioelectrical impedance analysis (BIA), or anthropometric measurements (e.g., circumferences).
• Study Design (S): randomized controlled trials (RCTs)

Exclusion criteria included studies involving individuals aged ≤18 years or ≥65 years, with BMI < 25 kg/m^2^, or with physical or cognitive disabilities that could directly compromise motor function. Studies were also excluded if the intervention was not adequately described, if no control or comparative exercise group was included, or if body composition was not assessed as a primary or secondary outcome.

### 2.4. Operational Definitions and Classification Criteria for Training Modalities

For this systematic review, we adopted operational definitions and minimum inclusion criteria to distinguish FT, RT and HIIT and to guide study selection. FT was defined as a structured intervention based on multi-joint, multi-segmental and multi-planar movements with intentional variation of load, velocity and task constraints, aimed at producing integrated and transferable adaptations (e.g., strength, power, endurance, balance, coordination, mobility and motor control) through the within-session integration of capacities, systematic progression and neuromuscular challenges that increase motor control demands [8]. RT was defined as a protocol whose primary stimulus is progressive overload to elicit gains in strength, power or hypertrophy, characterized by systematic manipulation of intensity (percentage of one-repetition maximum—%1-RM or equivalent), volume (sets × repetitions), frequency and rest intervals, with predominance of resistive exercises and emphasis on segmental or isolated stimuli. HIIT was defined as repeated cycles of high-intensity efforts (typically near-maximal or ≥85% maximal oxygen uptake—VO_2_max) interspersed with brief recoveries, emphasizing cardiorespiratory/metabolic stress and an explicit work–rest structure; when functional movements predominate but the primary objective is interval aerobic stress, the intervention was labeled HIIT-F [6,8].

A protocol was classified as FT only if it cumulatively met the following criteria: (1) predominance of multi-joint/multi-planar movement patterns; (2) intentional integration of at least two physical capacities within sessions (e.g., strength + balance); (3) a documented progression scheme (intensity, volume, or complexity); (4) inclusion of neuromuscular challenge beyond isolated resistance work (e.g., unilateral tasks, instability, velocity, or directional variation); (5) clear reporting of dose and monitoring (frequency, session duration, work–rest ratio, objective or validated intensity metrics); and (6) explicit justification or evidence distinguishing the program from conventional RT and from pure HIIT. To ensure transparency and reproducibility of the classification process, the fulfillment of these criteria by each included study is detailed in Supplementary Table S1.

Modal classification was performed by two independent reviewers who extracted session templates (warm-up, main set, cool-down), exercise lists with movement classification, time allocation per component, intensity metrics (heart rate—HR, %1RM, %VO_2_max, rated perceived exertion scale—RPE/Borg), work–rest ratios and progression schemes. Studies meeting all FT criteria were classified as FT; interventions in which ≥50% of session time comprised progressive resistive exercise without intentional capacity integration were classified as RT; interval-structured protocols with short, intense efforts and defined recoveries were classified as HIIT (or HIIT-F when functionally oriented); mixed interventions were labeled as CT and described by component proportion and sequencing. Studies failing to meet the FT criteria were included only in analyses corresponding to their assigned modality. This approach was adopted to minimize misclassification, enhance comparability across trials and enable modality-stratified quantitative syntheses.

### 2.5. Literature Search

A comprehensive literature search was conducted in January 2026 in the following databases: PubMed/MEDLINE, Embase, Web of Science, Cochrane Library, PEDro, and LILACS. Google Scholar was also searched to identify gray literature, with screening limited to the first 10 pages of results due to decreasing relevance. Reference lists of the included articles were also manually screened to identify potentially eligible articles. No language restrictions were applied.

The initial search strategy was developed for PubMed/MEDLINE using a combination of Medical Subject Headings (MeSH) and free text terms and was subsequently adapted to match the indexing systems and syntax of each database. Key search terms included: “Adult,” “Middle Aged,” “Overweight,” “Obesity,” “Functional Training,” “Functional Exercise,” “Functional Task Training,” “Multicomponent Training,” and “Body Composition”. Boolean operators AND and OR were used to combine terms and maximize search sensitivity. The full search strategies for all databases are available in Supplementary Material S2.

### 2.6. Studies Selection

Study selection was performed in two sequential and independent stages. First, two reviewers (ACAMB and LESS) screened the titles and abstracts retrieved from the databases using the predefined eligibility criteria. In the second stage, full texts of potentially relevant articles were reviewed to confirm inclusion.

The screening process was conducted using the Rayyan platform [14], which facilitates study organization, duplicate removal, and tracking of inclusion and exclusion decisions. Any disagreements between reviewers were resolved by a third reviewer.

### 2.7. Data Extraction

Data were extracted using a standardized form and included the following elements: author and publication year, study population, details of the intervention groups (total duration, session length, number of sets, rest intervals, weekly frequency, intensity, and specific exercises), assessment tools, body composition outcomes, main findings, and effect sizes when reported.

### 2.8. Assessment of Methodological Quality

The risk of bias of the included randomized controlled trials was assessed using the revised Cochrane Risk of Bias tool—RoB 2 [15], developed by Sterne JAC et al. (2019). This tool evaluates five domains: (1) bias arising from the randomization process; (2) bias due to deviations from intended interventions; (3) bias due to missing outcome data; (4) bias in measurement of the outcome; and (5) bias in selection of the reported result. Each domain was judged as “low risk of bias”, “some concerns”, or “high risk of bias”, leading to an overall risk of bias judgment for each study. Two reviewers (ACAMB and LESS) independently assessed study quality, with disagreements resolved by a third researcher. The results of the risk of bias assessment were presented using visual summaries and were considered in the interpretation of the findings.

### 2.9. Assessment of the Certainty of the Evidence

The certainty of the evidence was assessed using the GRADE system (Grading of Recommendations Assessment, Development and Evaluation), by outcome, considering the domains of risk of bias, inconsistency, indirectness, imprecision, publication bias, and other relevant considerations. Randomized controlled trials were initially classified as high-certainty evidence and downgraded according to established criteria.

For %BF, certainty assessment was based on the pooled estimate obtained from the meta-analysis, with imprecision evaluated according to the 95% confidence intervals. For the remaining body composition outcomes, which could not be quantitatively synthesized, certainty was assessed based on the narrative synthesis, considering the risk of bias of the included studies, consistency in the direction of effects, clinical and methodological heterogeneity, sample size, assessment methods, and the availability of comparable numerical data.

The summary of findings table was generated using GRADEpro GDT [16].

### 2.10. Data Synthesis

The data were synthesized descriptively, focusing on sample characteristics, intervention type, protocol duration and frequency, outcome measures, and principal findings. Extracted data were summarized in tables and narratively presented to provide a comprehensive overview of the available evidence regarding FT and body composition in adults with overweight or obesity.

A quantitative synthesis was performed when at least two studies reported comparable numerical data for the same outcome and comparison. %BF was the only outcome eligible for meta-analysis because two randomized controlled trials compared FT or HIIT-F with a non-exercising control group and reported post-intervention means, standard deviations, and sample sizes. Quantitative synthesis was restricted to this outcome because the remaining studies exhibited substantial clinical and methodological heterogeneity, particularly regarding comparator groups, outcome definitions, and data reporting formats, which precluded robust pooling of results.

The meta-analysis was conducted using Review Manager software (RevMan, version 5.4.1) [17]. %BF was analyzed as a continuous outcome, and pooled estimates were calculated using the mean difference because all included studies reported this outcome using the same unit of measurement. A random-effects model was adopted owing to clinical and methodological differences between studies, including intervention duration and body composition assessment methods.

Post-intervention values were used to calculate pooled estimates because change-score standard deviations and adjusted between-group estimates were not consistently reported across studies. Baseline comparability between intervention and control groups was verified before quantitative synthesis. Additionally, a sensitivity analysis was performed using the 20-week assessment point from Batrakoulis et al. [18] to evaluate the influence of follow-up duration on the pooled effect estimate.

## 3. Results

### 3.1. Study Selection

**Figure 1:**
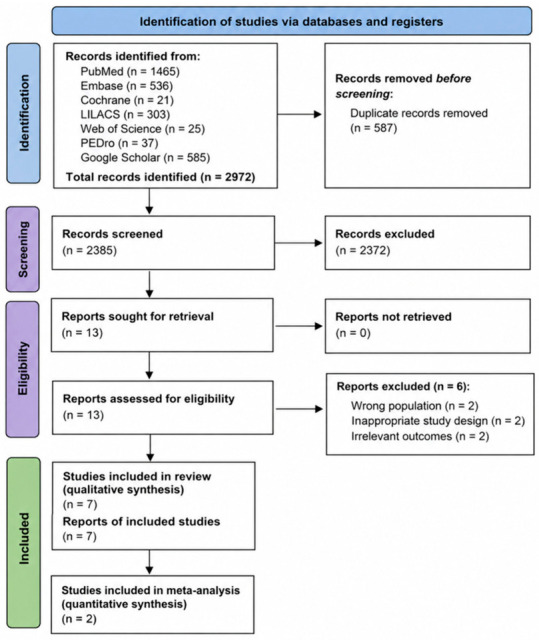
PRISMA flow diagram of the study selection process.

The initial search yielded 2972 records. After deduplication (*n* = 587), 2385 records were screened by title and abstract screening, resulting in 13 full-text articles assessed for eligibility. Seven studies met all inclusion criteria and were included in the final review. No additional studies were identified through manual searching. The study selection process is detailed in the PRISMA flowchart (Figure 1), which outlines the screening stages and reasons for exclusion.

### 3.2. Characteristics of Included Studies

Key characteristics of the included studies are summarized in Table 1. The seven RCTs included 296 participants, with sample sizes ranging from 18 [19] to 64 participants [20,21]. There was a predominance of female participants (86.1%), with only three studies including male participants [19,20,22].

The comparators varied across studies: RT programs [19,20], running-based HIIT [22], interdisciplinary interventions [23], and dietary restriction/intermittent fasting, either alone or combined with HIIT-functional (HIIT-F) [21]. Two studies compared FT alone with FT combined with another modality [10,18], with Batrakoulis et al. [18] also including a detraining period (20 weeks) to assess the maintenance of intervention effects.

The mean duration of interventions was 16.7 ± 13 weeks. Four studies had durations ≥ 12 weeks [20,21,22,23], while three lasted ≤ 9 weeks [10,19,20]. Most protocols included 3 weekly sessions, with session duration ranging from 25 to 60 minutes. Intensity was commonly monitored using an RPE/Borg, although some protocols used physiological markers such as %HR or %1RM. Training descriptions indicated the use of multi-joint circuits involving bodyweight and/or free weights, although substantial heterogeneity was noted across studies—with greater similarity between the protocols of Teixeira et al. [23] and Cao et al. [22].

Regarding outcome assessment methods, five studies used BIA [10,20,21,22,23], while two used DXA [18,19]. Most studies measured %BF and fat mass (FM); only one study assessed estimated visceral fat [22].

### 3.3. Effects of Functional Training on Body Composition in Adults with Excess Weight

Overall, the studies suggest a favorable direction of FT, either in isolation or combined with other interventions, on reducing total adiposity (FM and/or %BF) and, in some cases, on increasing fat-free mass (FFM) or LM. However, these effects were not consistent across studies and appeared to vary based on the type of comparator, intervention duration and content, and measurement methods (DXA vs. BIA), all of which limit direct comparisons and the estimation of standardized effect sizes (Table 2).

In young women with overweight, Sperlich et al. [10] compared a 9-week HIIT-F protocol using bodyweight circuits to a combined version that added one low-intensity, high-volume running session per week. Both interventions yielded similar results, reducing FM and increasing FFM with no significant between-group differences. BIA estimates indicated small to moderate improvements, suggesting that the addition of a continuous low-intensity aerobic component did not substantially enhance the effects of functional HIIT.

Batrakoulis et al. [18] evaluated women with excess weight undergoing 20 weeks of circuit-based FT (≥65% HR) and observed reductions in body weight and FM, along with increases in FFM, as measured by DXA. After a 20-week detraining period, these benefits were attenuated but not completely lost, indicating some persistence of adaptations of body composition in the medium term.

In contrast, Feito et al. [19] compared an 8-week combined training program (aerobic + machine-based resistance training) with whole-body HIIT-F (cross-training style) in adults with excess weight. No statistically significant between-group differences were observed for DXA-based body composition outcomes. However, within the HIIT-F group, there was a trend toward reduced %BF (*p* = 0.08) and a statistically significant increase in lower limb LM (*p* = 0.01). Therefore, these findings should be interpreted as within-group changes rather than evidence of superiority of HIIT-F over the comparator intervention.

In a 30-week study involving women with obesity, Teixeira et al. [23] reported that FT (3 times/week) did not yield significant changes in body mass, FM (absolute or relative), or FFM compared to interdisciplinary therapy and educational programs. BIA-based assessments showed no relevant differences, suggesting that FT alone may be insufficient to induce measurable body composition improvements in this population, thereby highlighting the potential need for integrated, multidisciplinary strategies. In middle-aged adults, Cavaggioni et al. [20] found that 6 weeks of circuit-based FT and RT (both 3 times/week) led to reductions in %BF, with no differences between modalities. Effect sizes were modest and consistent with the relatively short intervention duration, suggesting that both modalities may yield comparable short-term reductions in adiposity.

Cao et al. [22] explored two HIIT protocols in young adults with overweight. Both full-body HIIT-F and running-based HIIT (3×/week for 12 weeks) reduced body weight and %BF. However, HIIT-F led to a greater increase in LM (+1.623 kg; *p* < 0.001), while running-based HIIT was more effective in reducing estimated visceral fat. These findings indicate modality-specific adaptations, with functional protocols favoring muscle accretion and locomotor protocols favoring visceral fat reduction.

Finally, in women with obesity, Ameur et al. [21] showed that combining intermittent fasting with full-body HIIT-F (12 weeks) produced greater improvements in body composition—reduced FM and increased LM—compared to either intervention alone. Reported partial effect sizes (ηp^2^ = 0.33 for FM; ηp^2^ = 0.27 for LM) support a synergistic effect between energy restriction and high-intensity functional training, suggesting that integrated interventions enhance body recomposition.

### 3.4. Methodological Quality of the Included Studies

The risk of bias assessment revealed variability in methodological quality among the included studies (Table 3). None of the studies were classified as having a low overall risk of bias. Three studies were considered to have a high risk of bias [18,19,23], mainly due to problems related to a lack of outcome data and deviations from planned interventions.

The most frequent sources of bias were related to deviations from planned interventions and incomplete outcome data, often associated with the absence of intention-to-treat analysis and loss of participants. In contrast, bias in outcome measurement was consistently considered low risk in all studies, since body composition was assessed using objective methods.

Four studies were classified as having some concerns [10,20,21,22], mainly due to insufficient information on allocation concealment and a lack of clarity regarding pre-specified analysis plans. Overall, these findings indicate moderate methodological limitations in the included evidence base, which should be considered in the interpretation of the results of this review.

### 3.5. Certainty of Evidence

The certainty of the evidence was rated as very low for all evaluated outcomes. For %BF, the evidence was downgraded due to risk of bias, indirectness related to the use of different body composition assessment methods, and imprecision stemming from the small number of studies and participants. The meta-analysis included two studies with a total of 61 participants, demonstrating a reduction in %BF in favor of FT/HIIT-F. However, confidence in this estimate remains limited (Table 4).

For LM/FFM, FM, body weight, BMI, waist circumference/central adiposity, and visceral fat area, the certainty of the evidence was likewise rated as very low. These outcomes could not be quantitatively pooled due to heterogeneity in comparator groups, interventions, follow-up durations, assessment instruments, and incomplete availability of comparable data. Consequently, these findings were presented through narrative synthesis and should be interpreted with caution.

### 3.6. Quantitative Synthesis

Two studies were included in the meta-analysis of %BF [18,22], comprising a total of 61 participants. Batrakoulis et al. [18] compared FT with a non-exercising control group and assessed body composition using DXA. For the primary analysis, the 40-week assessment point was selected because it represented the end of the continuous training period. Cao et al. [22] compared HIIT-F with a non-exercising control group, with body composition evaluated by BIA, and post-intervention values at 12 weeks were used for quantitative synthesis.

**Figure 2:**
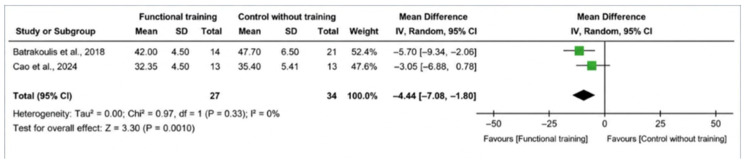
Forest plot for body fat percentage: FT/HIIT-F versus control without training. Squares represent the effect estimate of each study, with square size proportional to the study weight; horizontal lines represent the corresponding 95% confidence intervals; and the diamond represents the pooled effect estimate. MD: mean difference; CI: confidence interval; IV: inverse variance [18,22].

The pooled analysis demonstrated a significant reduction in %BF in favor of FT/HIIT-F compared with the control condition (MD = −4.44 percentage points; 95% CI −7.08 to −1.80; *p* = 0.001; I^2^ = 0%). Although statistical heterogeneity was not detected, these findings should be interpreted with caution given the small number of included studies and the use of different body composition assessment methods (Figure 2).

### 3.7. Sensitivity Analysis

**Figure 3:**
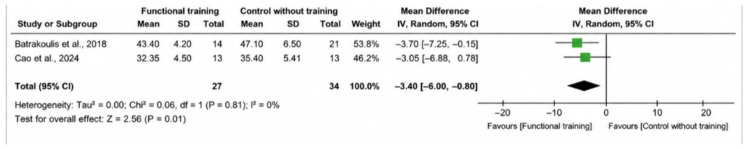
Sensitivity analysis for body fat percentage: Batrakoulis 20-week time point. Squares represent the effect estimate of each study, with square size proportional to the study weight; horizontal lines represent the corresponding 95% confidence intervals; and the diamond represents the pooled effect estimate. MD: mean difference; CI: confidence interval; IV: inverse variance [18,22].

A sensitivity analysis was conducted using the 20-week assessment point from Batrakoulis et al. [18], while retaining the 12-week post-intervention data from Cao et al. [22]. The pooled analysis demonstrated a significant reduction in %BF in favor of FT/HIIT-F compared with the non-exercising control group (MD = −3.40 percentage points; 95% CI −6.00 to −0.80; *p* = 0.01; I^2^ = 0%). The direction and statistical significance of the effect remained consistent with those observed in the primary analysis, indicating that the pooled estimate was not substantially influenced by the selection of the follow-up time point from Batrakoulis et al. [18] (Figure 3).

## 4. Discussion

This systematic review identified a limited number of high-quality studies investigating the effects of FT on body composition in individuals with excess weight. Traditionally, obesity diagnosis relies on the BMI, a simple and widely used anthropometric measure. However, despite its practicality for population-level assessment, BMI has recognized limitations in distinguishing between FM and LM. Therefore, complementary assessments such as waist-to-hip ratio and, when available, direct body composition techniques are recommended for a more accurate evaluation of adiposity [24].

Moreover, the body composition outcomes observed in the present review appear to be consistent with those reported for other exercise modalities commonly prescribed to adults with overweight or obesity. In a recent meta-analysis, Khalafi et al. [25] demonstrated that exercise-based interventions, including AT, HIIT, and CT programs, particularly when combined with dietary strategies, promoted significant reductions in body weight, BMI, body fat, and visceral adiposity. Similar results were observed by Wang et al. [6], whose network meta-analysis identified beneficial effects of AT, RT, CT, and HIIT on body composition outcomes in adults with overweight or obesity. In this context, the reductions in %BF observed following FT suggest that this modality may provide benefits comparable to those achieved with more established exercise approaches for the management of overweight and obesity, while offering the additional advantage of integrating multiple physical capacities within a single training session.

In this regard, a detailed analysis of body composition provides essential insights into the pathophysiological mechanisms associated with excess weight. Regarding LBM—defined as all body components excluding adipose tissue [26]—six of the studies analyzed reported favorable increases in LBM among participants undergoing FT interventions [10,18,21,22,23], although these findings were not uniformly observed across all studies and should be interpreted considering differences in study design and comparator conditions. In particular, Cao et al. [22] reported an increase in LBM among participants engaged in FT, whereas those performing running-based training exhibited a reduction. These findings are consistent with previous reports [3,25], suggesting that regular exercise incorporating resistance components may contribute to the preservation of LM, a major determinant of basal metabolic rate and a factor associated with the prevention of weight regain after fat loss.

Concerning FM, which encompasses both subcutaneous and visceral adipose tissue and represents a primary marker of lipid accumulation associated with cardiometabolic risk, the reviewed studies showed that FT generally reduces FM in individuals with overweight or obesity, particularly when combined with nutritional strategies [20,21,23]. In contrast, Feito et al. [19] did not observe significant reductions in FM, likely due to methodological limitations such as short intervention duration (8 weeks), small sample size (*n* = 18), and absence of nutritional control. Longer interventions, exemplified by Batrakoulis et al. (40 weeks) [18] and Teixeira et al. (30 weeks) [23], were among the studies reporting more favorable body composition outcomes. However, the substantial heterogeneity in intervention characteristics, comparator conditions, and co-interventions precludes determining the independent contribution of intervention duration to these findings. Likewise, studies incorporating nutritional components frequently reported favorable changes in adiposity; however, the available evidence does not allow the specific effects of dietary strategies to be distinguished from those of exercise interventions. Therefore, although longer interventions and combined exercise–nutrition approaches were often associated with more favorable body composition outcomes, causal relationships cannot be established, and these observations should be interpreted cautiously.

Across the included studies, a lack of standardization in FT session structure—particularly regarding neuromuscular stimuli, exercise selection, and load parameters—complicates the interpretation and replication of results. For example, Sperlich et al. [10] employed a high-intensity circuit protocol involving exercises such as burpees, single-leg squats, and push-ups, with 30-second rest intervals, whereas Batrakoulis et al. [18] combined bodyweight exercises with tools such as balance balls, battle ropes, and kettlebells. Despite these differences, most interventions maintained methodological consistency concerning duration (45–60 min) and frequency (three sessions per week) [10,18,19,20,21,23], which aligns with the recommendations of the Brazilian Association for the Study of Obesity and Metabolic Syndrome [27], suggesting 150–300 minutes of moderate-intensity activity per week and emphasizing resistance exercise to preserve LM.

Cao et al. [22] implemented shorter sessions (25 minutes), yet their findings remain valid: the Physical Activity Guidelines for Americans (2nd ed.) acknowledge that brief, regularly performed sessions at sufficient intensity can confer meaningful health benefits [28]. Consequently, both short- and long-format FT protocols may be effective provided that continuity, adequate intensity, and total weekly volume are preserved. This temporal flexibility may facilitate adherence among time-constrained individuals and may represent a practical characteristic of FT interventions in real-world settings.

Regarding body composition assessment, most studies used BIA to estimate FM and LBM [10,19,20,21,22,23]. Although BIA offers practicality, low cost, and portability, its accuracy may be compromised in individuals with obesity due to alterations in the ratio of intracellular to extracellular water, which affects the reliability of FM and FFM estimates. Since the technique is highly sensitive to total body water—and LM is composed of approximately 73% water—any condition that modifies fluid distribution, such as obesity, may introduce systematic errors in body composition assessment. Changes in hydration status and extracellular fluid distribution can influence impedance measurements and the prediction equations used by BIA devices, potentially affecting the estimation of FM and FFM. Consequently, individuals with high BMI may present overestimation of FFM when assessed by BIA [26,29].

Conversely, DXA remains the most reliable and clinically valid tool for assessing body composition, providing comprehensive data on FM, LM, bone mineral density, and visceral fat distribution, with minimal radiation exposure. DXA also offers a cost-effective alternative to imaging-based methods such as computed tomography or magnetic resonance, maintaining diagnostic precision while facilitating large-scale applicability [26].

An important limitation of the current evidence base is the marked predominance of female participants across the included studies. Women represented 100% of the sample in four studies [10,18,21,23] and 86.1% of participants in the remaining studies [19,20,22]. Consequently, the external validity of the present findings is limited, and their applicability to men should be interpreted with caution. Because body composition, hormonal regulation, substrate utilization, and physiological responses to exercise differ between sexes, the magnitude and direction of adaptations to FT may not be identical in men and women. For example, men generally present higher testosterone concentrations, greater FFM, and a larger proportion of visceral adipose tissue, whereas women typically exhibit higher estrogen concentrations, greater subcutaneous fat deposition, and distinct fatigue and endurance profiles, all of which may influence body composition responses to exercise interventions [30].

Additional factors may further contribute to the predominance of women in this field of research. Hormonal fluctuations across the female life course, particularly variations in estrogen and progesterone concentrations, influence muscle strength, cardiorespiratory fitness, and fat distribution [31]. None of the studies included explicitly controlled these variables, which may have introduced additional variability into the findings. Furthermore, sociocultural factors may also influence participation patterns in weight-management research. Women have historically been subjected to greater social pressure regarding body image, body weight, and thinness ideals, factors that have been proposed as possible influences on participation in weight-management interventions and related clinical research [32]. Although these factors may partially explain the predominance of women in the available evidence, they also highlight an important knowledge gap regarding the effects of FT in men. Future randomized controlled trials should prioritize a more balanced sex distribution and consider hormonal status in their analyses to improve the generalizability, applicability, and reproducibility of the evidence.

Another determinant of intervention success is participant adherence. Sustained engagement in structured exercise is essential for achieving the chronic physiological adaptations underlying weight management [33,34]. Adherence, however, depends on multiple psychosocial factors, including self-efficacy, social support, enjoyment, and integration into daily routines. While most studies reported adherence rates above 85%, only one failed to disclose adherence [20], and one reported a notably low rate (56%) due to personal and contextual factors such as pregnancy, surgery, or occupational changes [23]. Designing individualized, accessible, and engaging FT programs—responsive to participants’ schedules, preferences, and capabilities—may enhance long-term participation and consolidate the benefits of intervention [35].

Current evidence indicates that FT may contribute to reductions in %BF among adults with overweight or obesity, in line with benefits reported for other exercise modalities commonly employed in weight-management programs. However, confidence in these findings is limited by methodological constraints, small sample sizes, and heterogeneity in intervention design, comparator conditions, and body composition assessment methods, all of which restrict quantitative synthesis and reduce the generalizability of results.

Moreover, although all included interventions met the predefined FT criteria, they represented different manifestations along a continuum of functionality, ranging from traditional FT to HIIT-F and functionally oriented multicomponent programs. This diversity underscores that outcomes should not be attributed to a single intervention component but rather interpreted within the context of the different FT manifestations represented across the included studies.

Within this context, although most trials reported favorable effects of FT, only %BF could be pooled with sufficient methodological consistency. The meta-analysis suggests that FT/HIIT-F may be associated with reductions in %BF among adults with overweight or obesity and demonstrated a statistically significant mean reduction of −3.40 percentage points in favor of FT/HIIT-F. Despite the clinical relevance of this magnitude, the finding should be interpreted with caution given the limited number of included studies, small sample sizes, heterogeneity in intervention duration, differences in body composition assessment methods, and the very low certainty of the available evidence according to the GRADE assessment.

In particular, Batrakoulis et al. [18] employed DXA, whereas Cao et al. [22] used BIA. Although both methods report %BF, they differ in terms of validity, precision, and measurement assumptions. Furthermore, the included studies varied in methodological quality, comparator conditions, and outcome reporting procedures. Therefore, the pooled estimate should be interpreted as a limited quantitative synthesis rather than definitive evidence of effectiveness.

The quantitative synthesis was restricted to %BF and included only two studies. Other body composition outcomes, such as FM and FFM/LM, could not be pooled because of differences in comparator groups, intervention designs, outcome reporting formats, and incomplete availability of numerical data. Additionally, post-intervention values were used for quantitative synthesis because change-score standard deviations and adjusted between-group estimates were not consistently available across studies. Although baseline %BF was comparable between intervention and control groups, residual baseline differences cannot be completely excluded. Furthermore, the absence of statistical heterogeneity should not be interpreted as evidence of methodological homogeneity, as the meta-analysis comprised only two randomized controlled trials. These methodological limitations are reflected in the GRADE assessment, which rated the certainty of the evidence as very low for all body composition outcomes. Therefore, the current evidence should be regarded as preliminary and hypothesis-generating rather than conclusive.

Confirmatory evidence will depend on larger, high-quality randomized controlled trials employing standardized protocols, stratified analyses, validated body composition methods, and harmonized outcome reporting to enable more reliable pooled analyses.

## 5. Conclusions

This systematic review suggests that FT/HIIT-F may be associated with reductions in %BF among adults with overweight or obesity. However, the certainty of the available evidence was rated as very low, and the findings should be interpreted with caution due to the limited number of randomized controlled trials, small sample sizes, risk of bias, methodological heterogeneity, and differences in body composition assessment methods. For the remaining body composition outcomes, including FM, LM/FFM, body weight, BMI, central adiposity, and visceral fat area, the evidence remains uncertain because these outcomes could not be quantitatively synthesized and were evaluated primarily through narrative synthesis.

The available findings do not support causal conclusions and should not be generalized beyond the predominantly female populations represented in the included studies. Variability in intervention protocols, body composition assessment methods, and outcome reporting limits comparability across studies, while the predominance of female participants restricts the external validity of the findings. Consequently, the current evidence is more directly applicable to women with overweight or obesity.

The methodological limitations identified in the included studies should also be interpreted within the broader context of exercise-based research. Challenges related to intervention implementation, participant adherence, and body composition assessment are common in exercise science and are not exclusive to FT interventions. Nevertheless, these limitations reinforce the need for cautious interpretation of the available evidence.

Future randomized controlled trials should adopt more rigorous methodological designs, standardized intervention protocols, validated body composition assessment methods, and harmonized outcome reporting. In addition, studies with a more balanced sex distribution and sex-stratified analyses are needed to improve the certainty, generalizability, and clinical applicability of the evidence regarding the effects of FT on body composition in adults with overweight or obesity.

## Figures and Tables

**Table 1:** Sample characteristics and training protocols of the included articles.

References	Training Protocol							
	N	Age (Years)	Female (%)	BMI (kg/m^2^)	Time (Weeks)	Intervention	Intervention Characteristics	Exercises
Sperlich et al., 2017 [10]	19	23 ± 2	100	28.1 ± 2.7	9	HIIT-F (*n* = 11)	3×/week, Borg RPE Scale	Bodyweight HIIT circuit training
				28.3 ± 3.3		HIIT-F combined (*n* = 8)	3×/week, Borg RPE Scale	2 Resistance training sessions in a bodyweight-based HIIT circuit + 1 session of low-intensity, high-volume running
Batrakoulis et al., 2018 [18]	49	36.4 ± 4.4	100	28.2 ± 2.8	40	FT (*n* = 14)	38–56 min, 3×/week, ≥65% HR	Circuit consisting of 10–12 stations (exercises), with 20–40 s of execution (maximum repetitions) and 20–40 s of recovery intervals—using bodyweight and/or free weights
				29.1 ± 3.0		Training–Detraining (*n* = 14)	38–56 min, 3×/week, ≥65% HR	Circuit composed of 10–12 stations (exercises), with 20–40 s of execution (maximum repetitions) and 20–40 s of recovery intervals—using bodyweight and/or free weights
				29.6 ± 3.0		Control group (*n* = 21)	No intervention	No exercise
Feito et al., 2019 [19]	18	26.8 ± 5.5	67	30.5 ± 2.9	8	CT (*n* = 9)	50 min, 3×/week, 40–60% HR reserve, 50–75% 1RM	Two aerobic sessions (treadmill, rowing, elliptical, or cycling) + resistance training (CYBEX machine) + one additional aerobic session
						HIIT-F (*n* = 9)	60 min, 3×/week	CrossFit—full-body movement patterns + mobility + aerobic training (running/jump rope) + bodyweight exercises + weightlifting
Teixeira et al., 2020 [23]	44	39.7 ± 5.9	100	35.5 ± 2.8	30	FT (*n* = 14)	60 min, 3×/week, Borg RPE Scale	Aerobic training (treadmill or cycling) combined with resistance training (4 circuits with 8 stations—using bodyweight, free weights, and resistance bands)
						IT (*n* = 19)	60 min, 3×/week, Borg RPE Scale + nutritionist 1×/week + psychologist 1×/week + physiotherapy 1×/week	Aerobic training (treadmill or cycling) combined with resistance training (4 circuits with 8 stations—using bodyweight, free weights, and resistance bands)
						IE (*n* = 11)	Monthly lectures + WhatsApp group	No exercise
Cavaggioni et al., 2021 [20]	64	50.5 ± 10.4	17.18	37.3 ± 4.0	6	FT (*n* = 32)	45 min, 3×/week, Borg RPE Scale	Circuit training—3 sets (30 s of execution per set, 15 s of rest between exercises, 2 min of rest between sets)
		50.4 ± 10.7		37.7 ± 4.2		Conventional training (*n* = 32)	45 min, 3×/week, Borg RPE scale	Circuit training with bodyweight resistance exercises (30 s of work per set, 15 s of rest between exercises, 2 min of rest between sets, total volume: 3 sets)
Cao et al., 2024 [22]	38	22.1 ± 2.1	48.88	25.2 ± 1.0	12	HIIT-F (*n* = 13)	25 min, 3×/week	Four sets of full-body exercises (each set includes 4 × 30 s of exercise with 30 s of rest between exercises, and 1 min of rest between sets)
						HIIT-running (*n* = 12)	25 min, 3×/week	Four sets of treadmill running (each set includes 4 × 30 s of exercise with 30 s of rest between bouts, and 1 min of rest between sets)
						Control group (*n* = 13)	no intervention	No exercise
Ameur et al., 2024 [21]	64	32.1 ± 10	100	35.03 ± 3.8	12	IF + HIIT-F (*n* = 20)	45–55 min, 3×/week, Borg RPE Scale	8 sets of 8 exercises (aerobic and resistance) using bodyweight or free weights (20 or 30 s of work/10 s of rest)
						IF (*n* = 20)	Restricted diet	No exercise
						HIIT-F (*n* = 24)	45–55 min, 3×/week, Borg RPE Scale	8 sets of 8 functional exercises (aerobic and resistance) using bodyweight or free weights (20 or 30 s of work/10 s of rest)

Note: IE: Interdisciplinary Education; HR: Heart Rate; IF: Intermittent Fasting; CT: Combined Training; IT: Interdisciplinary Therapy; FT: Functional Training; HIIT: High-Intensity Interval Training; HIIT-F: Functional High-Intensity Interval Training; 1RM: One-repetition maximum; RT: Resistance training; RPE: Rated perceived exertion.

**Table 2:** Effects of functional training on body composition in adults with excess weight.

References	Assessment Model		Observed Outcomes
	Instrument	Evaluated Variables	
Sperlich et al., 2017 [10]	Four-electrode bioimpedance (Model 1609N; Tanita Corp., Tokyo, Japan)	Fat mass (kg)	HIIT-F: 40.0 ± 4.9 →* 38.1 ± 5.6 HIIT-F combined: 40.2 ± 4.8 → 39.2 ± 5.6 Significant within-group reduction in HIIT-F; no significant between-group difference
		Fat-free mass (%)	HIIT-F: 26.5 ± 2.4 → 27.4 ± 2.6 HIIT-F combined: 26.4 ± 2.0 → 26.9 ± 2.5 Significant within-group increase in both groups; no significant between-group difference
Batrakoulis et al., 2018 [18]	Whole-body dual-energy X-ray absorptiometry (Lunar Prodigy Advance, GE Lunar Healthcare Corp., Madison, WI, USA)	Fat mass (kg)	FT: 37.3 ± 6.7 → 31.1 ± 6.9 Training–Detraining: 36.3 ± 6.0 → 34.6 ± 7.3 Control group: 37.4 ± 6.5 → 38.6 ± 6.5 Significant reduction in fat mass was observed following FT. Significant time × intervention interaction favored FT compared with the control condition
		Fat-free mass (kg)	FT: 40.8 ± 4.1 → 42.3 ± 4.5 Training–Detraining: 41.9 ± 3.2 → 42.1 ± 3.6 Control group: 42.8 ± 7.2 → 42.3 ± 6.6 Significant within-group increase in fat-free mass following FT; significant time × intervention interaction favoring FT compared with the control condition
Feito et al., 2019 [19]	Dual-energy X-ray absorptiometry (DXA; Lunar iDXA, GE Healthcare, Madison, WI, USA)	Fat mass (kg)	CT: 35.6 ± 7.1 → −0.6 ± 1.5 (MD) HIIT-F: 49.0 ± 10.4 → 0.6 ± 1.2 (MD) No significant within-group changes No significant difference between groups
		Lean mass (kg)	CT: 46.5 ± 9.3 → 0.1 ± 1.6 (MD) HIIT-F: 35.6 ± 7.1 → −0.6 ± 1.5 (MD) No significant between-group difference; a significant within-group increase in lower-limb lean mass was observed in the HIIT-F
Teixeira et al., 2020 [23]	Maltron BF-906 bioelectrical impedance analysis (BIA)	Absolute Fat mass (kg)	FT: 40.6 ± 6.8 → 39.6 ± 6.4 IT: 43.6 ± 7.6 → 40.6 ± 7.6 IE: 44.3 ± 6.0 → 42.3 ± 8.6 Significant time effect; no significant time × intervention interaction. Significant reduction only in IT
		Absolute Fat-free mass (kg)	FT: 50.6 ± 6.1 → 49.9 ± 5.9 IT: 52.4 ± 6.8 → 50.9 ± 6.6 IE: 53.2 ± 5.4 → 51.9 ± 6.2 Significant time effect; no significant time × intervention interaction. Significant reductions in IT and IE
Cavaggioni et al., 2021 [20]	Bioelectrical impedance analysis (BIA 101–RJL Systems, Akern srl, Florence, Italy)	Body fat (%), Fat mass	Absolute pre–post values were not reported. Significant main effect of time for body fat percentage, with no significant group effect or time × intervention interaction
Cao et al., 2024 [22]	Bioelectrical impedance analysis (InBody 770, Biospace Co., Seoul, Republic of Korea)	Body Fat (%)	HIIT-F: 34.63 ± 4.98 → 32.35 ± 4.50 HIIT-running: 38.40 ± 4.76 → 36.38 ± 4.23 Control group: 35.23 ± 4.98 → 35.40 ± 5.41 Significant reductions in both HIIT-F and HIIT-running groups; significant time × intervention interaction; no changes in the control group
		Lean mass (kg)	HIIT-F: 28.08 ± 4.53 → 29.70 ± 5.11 HIIT-running:28.53 ± 5.43 → 27.49 ± 5.39 Control group:28.37 ± 4.93 → 28.75 ± 5.41 Significant time effect and time × intervention interaction; HIIT-F increased lean mass, whereas HIIT-running decreased lean mass.
Ameur et al., 2024 [21]	Bioelectrical impedance analysis (Tanita TBF-300; Tanita Corp., Tokyo, Japan)	Fat mass (kg)	IF-HIIT-F: 39.3 ± 9.2 → 31.0 ± 6.7 IF: 35.6 ± 7.9 → 31.6 ± 6.1 HIIT-F: 35.8 ± 8.7 → 32.9 ± 8.6 Significant reductions in all groups; significant between-group differences favoring IF-HIIT-F
		Fat-free mass (kg)	IF-HIIT-F: 23.3 ± 2.9 → 26.9 ± 3.2 IF: 25.1 ± 3.7 → 24.1 ± 3.41 HIIT-F: 22.1 ± 3.3 → 25.6 ± 3.2 Significant increases in IF-HIIT-F and HIIT-F; significant between-group differences favoring exercise-containing interventions

Note: CT: Combined Training; FT: Functional Training; HIIT: High-Intensity Interval Training; HIIT-F: Functional High-Intensity Interval Training; IE: Interdisciplinary Education; IF: Intermittent Fasting; IT: Interdisciplinary Therapy; Kg: Kilogram; MD: Mean difference. * Values presented before the arrow (→) indicate the mean ± standard deviation at baseline (pre-intervention), whereas values after the arrow represent the mean ± standard deviation following the intervention (post-intervention).

**Table 3:** Risk of Bias Assessment using the Risk of Bias 2.0 tool.

Author, Year	D1	D2	D3	D4	D5	Overall
Sperlich et al., 2017 [10]	SC	SC	SC	LR	SC	Some Concerns
Batrakoulis et al., 2018 [18]	LR	HR	HR	LR	SC	High Risk
Feito et al., 2019 [19]	SC	SC	HR	LR	SC	High Risk
Teixeira et al., 2020 [23]	SC	HR	HR	LR	SC	High Risk
Cavaggioni et al., 2021 [20]	LR	SC	SC	LR	SC	Some Concerns
Ameur et al., 2024 [21]	LR	SC	SC	LR	SC	Some Concerns
Cao et al., 2024 [22]	SC	SC	SC	LR	SC	Some Concerns

Note: LR: low risk of bias; SC: some concerns; HR: high risk of bias; D1: bias arising from the randomization process; D2: bias due to deviations from intended interventions; D3: bias due to missing outcome data; D4: bias in outcome measurement; D5: bias in selection of the reported result.

**Table 4:** Summary of findings and GRADE certainty assessment. Question: Functional training or high-intensity functional training compared with control without training for adults with overweight or obesity. Setting: Adults with overweight or obesity.

Outcome	Certainty Assessment							No. of Patients		Effect		Certainty	Importance
	No. of Studies	Study Design	Risk of Bias	Inconsistency	Indirectness	Imprecision	Other Considerations	FT/HIIT-F	Control Without Training	Relative (95% CI)	Absolute (95% CI)		
Body fat percentage (%) (post-intervention; DXA or BIA; follow-up range: 12 to 40 weeks) *	2	Randomized trials	Serious ^a^	Not serious	Serious ^b^	Serious ^c^	None	27	34	—	MD 4.44 fewer (7.08 fewer to 1.8 fewer)	⊕◯◯◯ Very low ^a,b,c^	Critical
Lean mass/fat-free mass	6	Randomized trials	Serious ^d^	Serious ^e^	Serious ^e^	Serious ^f^	None	0	0	—	See comment	⊕◯◯◯ Very low ^d,e,f^	Critical
Fat mass (kg)	6	Randomized trials	Serious ^g^	Serious ^h^	Serious ^h^	Serious ^e^	None	0	0	—	See comment	⊕◯◯◯ Very low ^e,g,h^	Critical
Body weight (kg)	5	Randomized trials	Serious ^h^	Serious ^e^	Serious ^h^	Serious ^h^	None	0	0	—	See comment	⊕◯◯◯ Very low ^e,h^	Important
Body mass index (kg/m^2^)	5	Randomized trials	Serious ^h^	Serious ^f^	Serious ^h^	Serious ^f^	None	0	0	—	See comment	⊕◯◯◯ Very low ^f,h^	Important
Waist circumference/central adiposity	4	Randomized trials	Serious ^i^	Serious ^i^	Serious ^a^	Serious ^f^	None	0	0	—	See comment	⊕◯◯◯ Very low ^a,f,i^	Important
Visceral fat area	1	Randomized trial	Serious ^d^	Not serious	Serious ^h^	Very serious ^f^	None	0	0	—	See comment	⊕◯◯◯ Very low ^d,f,h^	Important

Note: BIA: bioelectrical impedance analysis; CI: confidence interval; DXA: dual-energy X-ray absorptiometry; FT: functional training; GRADE: Grading of Recommendations Assessment, Development and Evaluation; HIIT-F: high-intensity functional training; MD: mean difference. * For body fat percentage, the effect estimate was derived from the meta-analysis of two randomized clinical trials comparing FT/HIIT-F with a non-exercising control group. For the other outcomes, no pooled estimate was calculated due to clinical and methodological heterogeneity and the incomplete availability of comparable numerical data. a. Downgraded one level due to risk of bias. One included study was judged as high risk of bias and the other presented some concerns, mainly related to missing outcome data, deviations from intended interventions, and lack of allocation concealment. b. Downgraded one level for indirectness because body composition was assessed using different methods, DXA and BIA, which are not equivalent in validity, precision, and measurement assumptions. c. Downgraded one level due to imprecision. The total sample size was small (*n* = 61), with only two included studies, and the confidence interval, although not crossing the line of no effect, was relatively wide. d. Downgraded one level due to risk of bias. The body of evidence included studies judged as high risk of bias and others with some concerns according to the RoB 2.0 tool. e. Downgraded one level due to inconsistency because the included studies had small sample sizes and variability in results. In addition, the absence of pooled estimates and confidence intervals limited the precision of the effect estimate, increasing uncertainty. f. Downgraded one level due to imprecision because available studies were based on small sample sizes and no pooled estimate was available. g. Most included studies presented some concerns in the RoB 2.0 assessment, mainly due to a lack of clear allocation concealment, absence of blinding of participants and personnel, and potential deviations from intended interventions. Given the behavioral nature of the intervention, performance bias is likely, which may have influenced outcomes. h. There was substantial variability in the direction and magnitude of effects across studies. While some trials reported reductions in fat mass, others showed minimal or non-significant changes. Additionally, differences in intervention protocols, duration, and co-interventions contributed to heterogeneity. i. Downgraded for risk of bias, inconsistency, indirectness, and imprecision. Central adiposity outcomes included waist circumference and related indices, such as waist-to-hip ratio and waist-to-height ratio, which were reported heterogeneously across studies and could not be pooled quantitatively. Superscript letters indicate the corresponding explanations for downgrading the certainty of evidence.

## Data Availability

Data supporting the findings of this study are available from the corresponding author upon reasonable request.

## Supplementary Materials

The following supporting information can be downloaded at: https://www.mdpi.com/article/10.3390/jfmk11030275/s1.

## Abbreviations

The following abbreviations are used in this manuscript:

AT	Aerobic Training
BIA	Bioelectrical Impedance Analysis
BMI	Body Mass Index
CT	Combined Training
DXA	Dual-energy X-ray Absorptiometry
FFM	Fat-Free Mass
FM	Fat Mass
FT	Functional Training
GRADE	Grading of Recommendations Assessment, Development and Evaluation
HIIT	High-Intensity Interval Training
HIIT-F	Functional High-Intensity Interval Training
HR	Heart Rate
IE	Interdisciplinary Education
IF	Intermittent Fasting
IT	Interdisciplinary Therapy
LM	Lean Mass
PRISMA	Preferred Reporting Items for Systematic Reviews and Meta-Analyses
PROSPERO	International Prospective Register of Systematic Reviews
RCTs	Randomized Controlled Trials
RoB 2	Revised Cochrane Risk of Bias Tool 2
RPE	Rated Perceived Exertion
RT	Resistance Training
VO_2_max	Maximal Oxygen Uptake
WHO	World Health Organization
%BF	Body Fat Percentage
%HR	Percentage of Heart Rate
%1RM	Percentage of One-Repetition Maximum
%VO_2_max	Percentage of Maximal Oxygen Uptake
