# Supplementary material for: Effects of Functional Training on Body Composition in Adults with Overweight or Obesity: A Systematic Review and Meta-Analysis of Randomized Clinical Trials
Source: J Funct Morphol Kinesiol. 2026 Jul 17;11(3):275. doi: 10.3390/jfmk11030275 (PMC13398009; doi:10.3390/jfmk11030275)
Supplement: Supplementary file 1 [file jfmk-11-00275-s001.zip › SUPPLEMENTARY MATERIAL S1.pdf]

Table S1. Classification of the Included Studies According to the Predefined Functional Training Eligibility Criteria.

Study	C1	C2	C3	C4	C5	C6	Overall Decision	Classification
Sperlich *et al.*, 2017 [10]	✓	✓	✓	✓	✓	✓	Eligible	HIIT-F
Batrakoulis *et al.*, 2018 [18]	✓	✓	✓	✓	✓	✓	Eligible	FT
Feito *et al.*, 2019 [19]	✓	✓	✓	✓	✓	✓	Eligible	HIIT-F
Teixeira *et al.*, 2020 [23]	✓	✓	✓	✓	✓	✓	Eligible	FT
Cavaggioni *et al.*, 2021 [20]	✓	✓	✓	✓	✓	✓	Eligible	FT
Cao *et al.*, 2024 [22]	✓	✓	✓	✓	✓	✓	Eligible	HIIT-F
Ameur *et al.*, 2024 [21]	✓	✓	✓	✓	✓	✓	Eligible	HIIT-F

Note: FT: Functional Training; HIIT-F: Functional High-Intensity Interval Training;

Eligibility criteria:

C1 = Predominance of multi-joint and/or multi-planar movement patterns.

C2 = Intentional integration of at least two physical capacities within training sessions.

C3 = Presence of a documented progression scheme (intensity, volume, complexity, or training load).

C4 = Inclusion of neuromuscular challenges beyond isolated resistance exercises.

C5 = Clear reporting of exercise dose and monitoring procedures (frequency, session duration, work-to-rest ratio, and/or validated intensity metrics).

C6 = Explicit justification or evidence distinguishing the intervention from conventional resistance training and pure HIIT.

Note: Studies were classified as eligible when all six predefined criteria were satisfied.
