# Supplementary material for: Effects of Functional Training on Body Composition in Adults with Overweight or Obesity: A Systematic Review and Meta-Analysis of Randomized Clinical Trials
Source: J Funct Morphol Kinesiol. 2026 Jul 17;11(3):275. doi: 10.3390/jfmk11030275 (PMC13398009; doi:10.3390/jfmk11030275)
Supplement: Supplementary file 1 [file jfmk-11-00275-s001.zip › SUPPLEMENTARY MATERIAL S2 - SEARCH STRATEGY.pdf]

SUPPLEMENTARY MATERIAL S2 - SEARCH STRATEGY

SOURCES	TERMS	Total / DATA
**Pubmed**	(("adult"[MeSH Terms] OR "adult"[All Fields] OR "adults"[All Fields] OR "adult s"[All Fields] OR ("adulto"[All Fields] OR "adultos"[All Fields]) OR ("middle aged"[MeSH Terms] OR ("middle"[All Fields] AND "aged"[All Fields]) OR "middle aged"[All Fields]) OR ("Meia"[All Fields] AND ("idade"[All Fields] OR "idades"[All Fields]))) AND ("body composition"[MeSH Terms] OR ("body"[All Fields] AND "composition"[All Fields]) OR "body composition"[All Fields] OR ("Composicao"[All Fields] AND ("corporal"[All Fields] OR "corporals"[All Fields]))) AND ("obeses"[All Fields] OR "obesity"[MeSH Terms] OR "obesity"[All Fields] OR "obese"[All Fields] OR "obesities"[All Fields] OR "obesity s"[All Fields] OR ("obesidad"[All Fields] OR "obesidade"[All Fields]) OR ("overweight"[MeSH Terms] OR "overweight"[All Fields] OR "overweighted"[All Fields] OR "overweightness"[All Fields] OR "overweights"[All Fields]) OR "Sobrepeso"[All Fields]) AND ((("functional"[All Fields] OR "functional s"[All Fields] OR "functionalities"[All Fields] OR "functionality"[All Fields] OR "functionalization"[All Fields] OR "functionalizations"[All Fields] OR "functionalize"[All Fields] OR "functionalized"[All Fields] OR "functionalizes"[All Fields] OR "functionalizing"[All Fields] OR "functionally"[All Fields] OR "functionals"[All Fields] OR "functioned"[All Fields] OR "functioning"[All Fields] OR "functionings"[All Fields] OR "functions"[All Fields] OR "physiology"[MeSH Subheading] OR "physiology"[All Fields] OR "function"[All Fields] OR "physiology"[MeSH Terms]) AND ("education"[MeSH Subheading] OR "education"[All Fields] OR "training"[All Fields] OR "education"[MeSH Terms] OR "train"[All Fields] OR "train s"[All Fields] OR "trained"[All Fields] OR "training s"[All Fields] OR "trainings"[All Fields] OR "trains"[All Fields])) OR ("Treinamento"[All Fields] AND "Funcional"[All Fields]) OR (("multicomponent"[All Fields] OR "multicomponents"[All Fields]) AND ("education"[MeSH Subheading] OR "education"[All Fields] OR "training"[All Fields] OR "education"[MeSH Terms] OR "train"[All Fields] OR "train s"[All Fields] OR "trained"[All Fields] OR "training s"[All Fields] OR "trainings"[All Fields] OR "trains"[All Fields])) OR ("Treinamento"[All Fields] AND "multicomponente"[All Fields]) OR "Dual task exercises"[All Fields] OR ("exercicios"[All Fields] AND ("drug effects"[MeSH Subheading] OR ("drug"[All Fields] AND "effects"[All Fields]) OR "drug effects"[All Fields] OR "de"[All Fields]) AND "dupla"[All Fields] AND "tarefa"[All Fields]) OR (("functional"[All Fields] OR "functional s"[All Fields] OR "functionalities"[All Fields] OR "functionality"[All Fields] OR "functionalization"[All Fields] OR "functionalizations"[All Fields] OR "functionalize"[All Fields] OR "functionalized"[All Fields] OR "functionalizes"[All Fields] OR "functionalizing"[All Fields] OR "functionally"[All Fields] OR "functionals"[All Fields] OR "functioned"[All Fields] OR "functioning"[All Fields] OR "functionings"[All Fields] OR "functions"[All Fields] OR "physiology"[MeSH Subheading] OR "physiology"[All Fields] OR "function"[All Fields] OR "physiology"[MeSH Terms]) AND ("exercise"[MeSH Terms] OR "exercise"[All Fields] OR "exercises"[All Fields] OR "exercise therapy"[MeSH Terms] OR ("exercise"[All Fields] AND "therapy"[All Fields]) OR "exercise therapy"[All Fields] OR	1465<br><br>10th Jan, 2026

	"exercising"[All Fields] OR "exercise s"[All Fields] OR "exercised"[All Fields] OR "exerciser"[All Fields] OR "exercisers"[All Fields])) OR ("Exercicio"[All Fields] AND "Funcional"[All Fields]) OR "Functional Task Exercise"[All Fields] OR ("Exercicio"[All Fields] AND ("drug effects"[MeSH Subheading] OR ("drug"[All Fields] AND "effects"[All Fields]) OR "drug effects"[All Fields] OR "de"[All Fields]) AND "tarefa"[All Fields] AND "Funcional"[All Fields]) OR "Functional Task Training"[All Fields] OR ("Treinamento"[All Fields] AND ("drug effects"[MeSH Subheading] OR ("drug"[All Fields] AND "effects"[All Fields]) OR "drug effects"[All Fields] OR "de"[All Fields]) AND "tarefas"[All Fields] AND "funcionais"[All Fields]))) AND (clinicaltrial[Filter])	
**Embase**	('adult'/exp OR adult OR middle) AND ('aged'/exp OR aged) AND ('body'/exp OR body) AND composition AND ('obesity'/exp OR obesity OR 'overweight'/exp OR overweight) AND (((functional AND ('training'/exp OR training) OR multicomponent) AND ('training'/exp OR training) OR 'dual task exercises' OR functional) AND ('exercise'/exp OR exercise) OR 'functional task exercise' OR 'functional task training')	536<br><br>12th Jan, 2026
**Cochrane**	(adult OR adults OR adulto OR adultos OR "middle aged" OR "middle age" OR "meia idade")<br>AND<br>("body composition" OR "composição corporal" OR "composicao corporal")<br>AND<br>(obesity OR obese OR obesidade OR obeso OR obesos OR overweight OR sobrepeso)<br>AND<br>("functional training" OR "treinamento funcional" OR "multicomponent training" OR "treinamento multicomponente" OR "dual task exercises" OR "exercícios de dupla tarefa" OR "exercicios de dupla tarefa" OR "functional exercise" OR "exercício funcional" OR "exercicio funcional" OR "functional task exercise" OR "exercício de tarefa funcional" OR "exercicio de tarefa funcional" OR "functional task training" OR "treinamento de tarefas funcionais")	21<br><br>22th Janr, 2026
**LILACS (via BVS)**	((Adult OR Adulto OR Middle aged OR Meia idade) AND (Body composition OR Composição corporal) AND (Obesity OR Obesidade OR Overweight OR Sobrepeso) AND (Functional training OR Treinamento funcional OR Multicomponent training OR Treinamento multicomponente OR "Dual task exercises" OR "Exercícios de dupla tarefa" OR Functional Exercise OR Exercício Funcional OR "Functional Task Exercise" OR "Exercício de Tarefa Funcional" OR "Functional Task Training" OR "Treinamento de Tarefas Funcionais"))	303<br><br>24th Jan, 2026
**Web of Science**	(adult* OR "middle aged") AND "body composition" AND (obesity OR obese OR overweight) AND ("functional training" OR "multicomponent training" OR "dual task exercise" OR "functional exercise" OR "functional task training")	25<br><br>24th Jan, 2026

**PEDro**	obese* functional training* body composition*	37<br><br>29th Jan, 2026
**Google Scholar**	"treinamento funcional" AND obesidade AND adultos AND "composição corporal"	585<br><br>30th Jan, 2026
**Total**		2972

.
